# Advancements in Plant and Microbe-Based Synthesis of Metallic Nanoparticles and Their Antimicrobial Activity against Plant Pathogens

**DOI:** 10.3390/nano10061146

**Published:** 2020-06-11

**Authors:** Md. Arshad Ali, Temoor Ahmed, Wenge Wu, Afsana Hossain, Rahila Hafeez, Md. Mahidul Islam Masum, Yanli Wang, Qianli An, Guochang Sun, Bin Li

**Affiliations:** 1 State Key Laboratory of Rice Biology and Ministry of Agriculture Key Lab of Molecular Biology of Crop Pathogens and Insects, Institute of Biotechnology, College of Agricultural and Biotechnology, Zhejiang University, Hangzhou 310058, China; alibau201@gmail.com (M.A.A.); temoorahmed248@gmail.com (T.A.); afsana_07@yahoo.com (A.H.); rahila.impp@gmail.com (R.H.); an@zju.edu.cn (Q.A.); 2 Rice Research Institute, Anhui Academy of Agricultural Sciences, Hefei 230001, China; 3 Department of Plant Pathology and Seed Science, Sylhet Agricultural University, Sylhet 3100, Bangladesh; 4 Department of Plant Pathology, Bangabandhu Sheikh Mujibur Rahman Agricultural University, Gazipur 1706, Bangladesh; masum@bsmrau.edu.bd; 5 State Key Laboratory for Managing Biotic and Chemical Threats to the Quality and Safety of Agro-Products, Zhejiang Academy of Agricultural Sciences, Hangzhou 310021, China; sungc01@sina.com

**Keywords:** green synthesis, microorganisms, plant extracts, metallic nanoparticles, plant pathogens

## Abstract

A large number of metallic nanoparticles have been successfully synthesized by using different plant extracts and microbes including bacteria, fungi viruses and microalgae. Some of these metallic nanoparticles showed strong antimicrobial activities against phytopathogens. Here, we summarized these green-synthesized nanoparticles from plants and microbes and their applications in the control of plant pathogens. We also discussed the potential deleterious effects of the metallic nanoparticles on plants and beneficial microbial communities associated with plants. Overall, this review calls for attention regarding the use of green-synthesized metallic nanoparticles in controlling plant diseases and clarification of the risks to plants, plant-associated microbial communities, and environments before using them in agriculture.

## 1. Introduction

Nanotechnology is an emerging field of science with a wide range of applications in various areas including medicine and agriculture. In agriculture, nanotechnology can be exploited by the use of natural resources in the conservation, production and protection of crops and livestock [1]. Recently, biosynthesis of nanoparticles (NPs) or green synthesis of NPs has received much attention due to the biocompatibility, low toxicity, and eco-friendly nature of the process and NP products [2]. The use of biological materials, such as bacteria, yeast, mold, microalgae and plant extracts, to synthesize NPs has some advantages like less energy consumption and moderate technology without using toxic chemicals [3,4]. The application of nanotechnology in plant disease control is just emerging [5]. NPs can be used directly or as carriers of various pesticides for plant protection. Most of the studies have been done in laboratory conditions [6]. It is crucial for us to know the effects on plants, microbes associated with the plants in fields and overall ecosystems before the application of green-synthesized NPs in plant disease management.

## 2. Green Synthesis of Metallic Nanoparticles

Green synthesis of NPs is a cost-effective and eco-friendly technique that does not use toxic chemicals. This technique employs a number of reducing and stabilizing agents like microbes, plants and other natural resources to produce NPs for sustainable in manner [7,8]. The green synthesis of NPs has gained much attention due to it being eco-friendly, cost effective and highly stable [9]. Several studies have reported the production of NPs using plants and microorganisms [10,11]. The green synthesis methods of NPs are diversified, but organisms or their extracts are simply reacted with a metallic salt and then biological reduction is carried out to convert the metal to NPs. The produced NPs are readily available to use after proper characterization [10,12].

**Figure 1:**
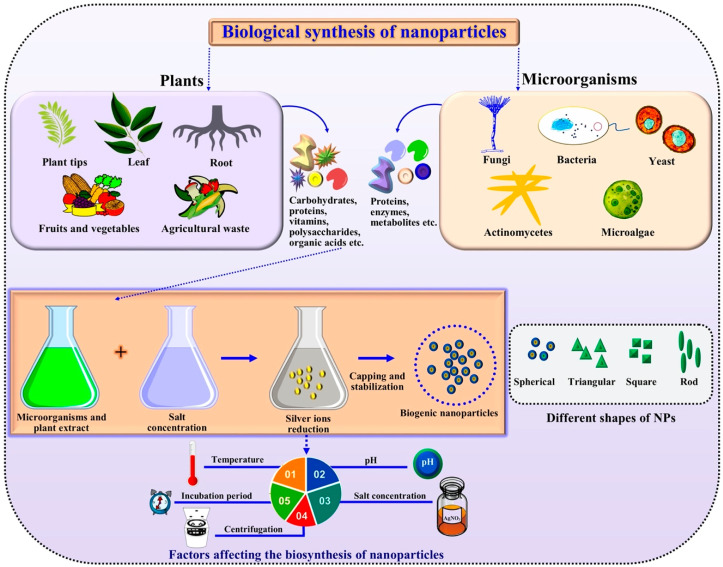
Generalized schematic representation of green synthesis of metallic nanoparticles (NPs).

Microbe-mediated synthesis of NPs is a green approach that utilizes bacteria, fungi, viruses and their products for the production of NPs. These microbes provide templates for synthesis and organization of well-defined, structured NPs [13,14]. In comparison to microbial synthesis as a potential technique, plants can be used in convenient manner for NPs production. The synthesis of NPs can be scaled up easily by using plant extracts. In addition, the plant extracts can reduce metallic ions more quickly than microbes and produce stable metallic NPs [10,15]. In plants extracts, many compounds like polysaccharides, proteins, amino acids, organic acids and phytochemicals like polyphenols, flavonoids, terpenoids, alkaloids, tannins, and alcoholic substances are present that can reduce and stabilize the NPs [12,16]. A generalized schematic representation of green synthesis of NPs is shown in Figure 1.

### 2.1. Microbe-Based Synthesis

The green synthesis mediated by microbes has been raised as an alternative method of NPs design and development [17,18]. Microbes can be used as safe and cheap tools for synthesis of metallic NPs like gold, silver, copper, zinc, titanium, palladium, and nickel. The synthesis of NPs can be carried out both extracellularly and intracellularly using microbes [19]. For extracellular synthesis, the culture filtrate is collected by centrifugation and mixed with an aqueous metallic salt solution. Synthesis of NPs is monitored by the color change of the mixed solution. For example, the light yellow to dark brown color is an indicator of synthesis of silver NPs (AgNPs) [8,20]. For intracellular synthesis, the biomass is washed thoroughly with sterile water after culturing microorganisms under optimum growth conditions and incubated with metal ion solution. As mentioned above, the color change serves as an indicator of NPs synthesis. Then NPs are collected by ultra sonication, centrifugation and washing [21]. Here, we review various metallic NPs synthesis through the utilization of microorganisms.

A diverse group of bacteria are living in soil, water, plants and animals. They can live in various soil pH, salinity, temperature and nutrient conditions. In aquatic environment, bacteria can be found in normal to highly saline water in deep-sea and even in the ice with a freezing temperature. Some of them can be occurred in heavily contaminated or hyper accumulated soils and plants. *Pseudomonas stutzeri* and *Pseudomonas aeruginosa* can survive even in high concentrated metal ion conditions [22,23]. *Thiobacillus ferrooxidans*, *T. thiooxidans* and *Sulfolobus acidocaldarius* can reduce ferric to the ferrous ion while living on elemental sulfur as an energy source [24]. Therefore, bacteria possess their own mechanisms by which they can survive and uptake nutrients for their growth and multiplication. They can reduce the metallic substances and utilize energy for themselves. Bacteria are evolving many defense mechanisms like sequestration intracellularly, pumping efflux, changing concentration of metal ions and precipitation extracellularly to overcome various stresses [25]. These types of mechanisms of bacteria can be applied in the green synthesis of NPs.

Recently, bacterial strains belonging to *Acinetobacter calcoaceticus*, *Bacillus amyloliquefaciens*, *Bacillus megaterium*, *Bacillus licheniformis*, *Escherichia coli*, *Lactobacillus sp.* and *Pseudomonas stutzeri* have been used in for the biosynthesis of AgNPs [26]. Silver NPs can be produced by both intracellular and extracellular biosynthesis and these NPs have shown antimicrobial activity against many pathological organisms [27]. The culture supernatant of *Pseudomonas rhodesiae* was incubated with AgNO_3_ solution and AgNPs were synthesized. A clear surface plasmon resonance peak at 420–430 nm in the range of 350–450 nm was confirmed as a featured peak of AgNPs by UV–Visible spectroscopy. The reduction of Ag^+^ and stabilization of the AgNPs was identified by Fourier-transform infrared spectroscopy. Transmission electron microscopy (TEM) and scanning electron microscopy (SEM) were performed to measure the size of the AgNPs synthesized with supernatant of *P. rhodesiae*. The AgNPs were generally spherical and uniform with a range of 20–100 nm in diameter. X-ray diffraction analysis was used to observe the crystalline nature of the *P. rhodesiae* mediated AgNPs [8]. An extracellular biosynthesis of AgNPs was carried out using *B. cereus* SZT1, isolated from wastewater-contaminated soil. The AgNPs were spherical shapes and their particle size ranged from 18 to 39 nm [18]. The culture filtrate of endophytic *Pseudomonas poae* strain CO was used to synthesize AgNPs with the size of 19.8–44.9 nm [20]. The AgNPs were biosynthesized using the culture supernatant of *Stenotrophomonas* sp. BHU-S7, which was isolated from agricultural farm soil [28]. The synthesis of gold NPs (AuNPs) using *Bacillus subtilis* isolated from Hatti Gold Mine was reported in a study. The microorganisms isolated from gold mine might be highly resistant to gold ions toxicity and could be used to synthesize AuNPs efficiently. The synthesis of ultra-small palladium and platinum NPs were done by using *Shewanella loihica* PV-4 within the size range of 2–7 nm [29]. *Ochrobactrum* sp. was used to synthesize tellurium NPs and this strain might serve as an effective nanofactory to convert the toxic tellurite oxyanions into useful NPs [30].

In addition to the isolation of bacteria from terrestrial environments some marine bacterial cultures have been utilized as nanofactories for synthesis of NPs. A novel bacterium *Stenotrophomonas* was used for green synthesis of AgNPs and AuNPs. Here, the secretory proteins with low molecular weight present in the supernatant play a key role for biosynthesis of AgNPs and AuNPs [31]. Another marine strain, *Kocuria flava*, was able to synthesize copper NPs with a size of 5 to 30 nm [32].

In a previous study, AgNPs were synthesized from *Pseudomonas stutzeri* AG259 through the process involving NADH-dependent reductase enzyme which provides electrons to oxidize NADH to NAD^+^. The donation of electron from NADH causes the bioreduction of Ag ions to AgNPs [33]. *Pseudomonas aeruginosa* SM1 can synthesize various NPs intracellularly, such as Ag, Fe, Co, Ni, Li, Pd, Pt and Rh NPs [34]. Moreover, some researchers have shown the synthesis of NPs without involving biological enzymes. For example, dead or inactive cells of *Corynebacterium glutamicum* were used to synthesize AgNPs. A large amount of reduction was found on the surface of the inactive cells resulting in the formation of AgNPs with irregular shape and size of 5 to 50 nm [35]. Zinc oxide NPs are also promising as antimicrobial agents, drug delivery and bioimaging probes in next-generation biological applications. Zinc oxide NPs were synthesized using a bacterium *Aeromonas hydrophila* in simple and cost-effective method. The crystalline nature of the NPs was observed by atomic force microscopy (AFM), which showed that the NPs were spherical and oval with an average size of 57.72 nm [36].

The conversion of metallic ions into NPs through reduction is dependent on functional groups of biomolecules present in the organisms which induce biomineralization, and other environmental factors, such as pH, media composition, concentration of metallic salts and temperature [33]. The size, shape and composition can be highly determined by these environmental factors [37]. For example, at the optimum growth temperature of 20 °C, spherical AgNPs were produced with an average diameter of 2–5 nm using *Morganella psychrotolerans*, while at 25 °C, a mixture of triangular and hexagonal nanoplates along with spherical NPs were obtained [38].

Actinomycetes, a group of filamentous bacteria, are known for their metabolic versatility. These bacteria can survive in stressful environmental conditions by using the bioactive potentials [39]. Actinomycetes consist of a significant composition of the microbial population in soils and produce extracellular enzymes to decompose materials. Their enzymes have received more attention than enzymes from other sources due to their high stability and uncommon substrate specificity. These are found in extreme habitats and produce enzymes with high commercial value [40]. Among the 22,000 discovered microbial secondary metabolites, 70% are from actinomycetes while two-thirds of them are originated from the genus *Streptomyces* [41]. Both extracellular and intracellular synthesis of NPs can be undertaken, but extracellular synthesis is a popular method and has been used commercially in various fields. Biomass extracts of *Streptomyces zaomyceticus* Oc-5 and *Streptomyces pseudogriseolus* Acv-11 were used for synthesis of copper oxide NPs (CuONPs). Green synthesized CuONPs were with surface plasmon resonance absorption band at 400 nm, crystalline, spherical with an average size of 78 nm and 80 nm for strain Oc-5 and Acv-11, respectively [42]. In another study, the free-biomass filtrates with metabolites from three endophytic actinomycetes of *Streptomyces capillispiralis* Ca-1, *Streptomyces zaomyceticus* Oc-5, and *Streptomyces pseudogriseolus* Acv-11 served as biocatalysts for green synthesis of AgNPs [43]. An actinobacteria *Rhodococcus* sp. was used to reduce aqueous AgNO_3_ for the green synthesis of AgNPs [44]. The extracellular synthesis of gold (Au) NPs was carried out using culture supernatant of soil isolated *Streptomyces griseoruber* with a size 5–50 nm [45]. The green synthesized metallic NPs show higher antimicrobial potentials than conventionally synthesized NPs because some biomolecules act as capping and stabilizing agents during synthesis of the NPs [14].

Fungi are excellent sources of many bioactive compounds that can be utilized in various sectors. The microscopic filamentous fungi (ascomycetes and imperfect fungi) and other fungal species are reported to produce about 6400 bioactive compounds [46]. These microorganisms possess tolerance to the heavy metals and are capable of internalizing as well as bioaccumulating the metals. So, these organisms have been used for reduction and stabilization during the synthesis of NPs. Moreover, large-scale cultivation of fungi is very easy and can be used to synthesize NPs with uniform shape and size [47,48,49,50]. Fungi are more convenient compared to other microbes due to their production of high quantities of enzymes and proteins for NPs synthesis [51,52]. The fungi mediated synthesis of NPs can be extracellular or intracellular [53]. For extracellular synthesis, the aqueous culture filtrates consisting of biomolecules are added to metal precursor, and free NPs are formed in the dispersion. This is a commonly used method, as no techniques are needed to get cell-free NPs [49,54,55,56]. During intracellular synthesis, a metal precursor is added to the mycelial culture and internalized in the biomass followed by the extraction of NPs. The extraction of the NPs is performed to disrupt the biomass by chemical treatment, centrifugation, and filtration and then release the synthesized NPs [57,58,59].

The fungal synthesis of metallic NPs is dependent on culture conditions. In a previous study, the culture conditions of *Trichothecium* sp. reduced Au ions resulting extracellular NPs synthesis but produced NPs intracellularly when cultured with agitations. The possible mechanism involved here is the non-agitation condition, but not the agitation condition, enhancing the release of enzymes and proteins [60]. The desired characteristics of NPs from different fungal species can be obtained through the adjustment of some factors like temperature, agitation, light, and culture and synthesis times. These parameters should be maintained during the fungal culture and NPs synthesis to control of the size and shape of NPs [61]. It was also found that differences in pH, temperature, culture medium, biomass quantity and concentration of the metal precursor can be used to determine physicochemical characteristics of NPs [58,61,62,63]. AgNPs were synthesized by using the filtrate of *Rhizopus stolonifer* with NPs size of 2.86, 25.89, and 48.43 nm under the temperature regime of 40, 20, and 60 °C, respectively, but not at 10 or 80 °C. At a very low or high temperature, enzymes and active molecules may be denatured or inactivated which are needed in AgNPs biogenesis [64].

Husseiny et al. reported the biosynthesis of AgNPs using *Fusarium oxysporum* and reported the effect of substrate concentration and incubation temperature [65]. Most of the AgNPs were smaller at 50 °C and, at higher temperature, particle size increased. The amount of biomass played a key role in synthesis or complete reduction of Ag^+^ to Ag^0^. The optimum weight of fungal biomass was 11 g for the smallest particle size. For the AgNPs synthesis by *F. oxysporum*, pH was found to be an important factor and the smallest size particles were obtained at pH 6. Due to the lower pH, protein structure might be affected or denatured and its potential may have been lost; thus, NP size was found to be large [66]. In alkaline conditions, the catalyzing activity of reductase enzyme for the synthesis might be gradually deactivated, and reduced synthesis and increase in size of the particles at higher pH. A similar phenomenon was observed during AgNPs synthesis using *Penicillium fellutanum* [67]. A seven-day old fungus used as a young culture for 72 h was better than a fifteen-day old culture for s similar incubation time in the case of AgNPs [65].

Yeast cells act as one of the most important agents for bioremediation of heavy metals. Yeasts are easily cultured in low-cost media and capable of removing various heavy metals. Yeasts have the adaptive capacity to extreme environmental conditions like pH, temperature and high concentrated organic and inorganic contaminants. Most of the available studies concern Ascomycota such *Saccharomyces cerevisiae*, *Schizosaccharomyces pombe* and *Candida* sp. Yeasts may have evolved some mechanisms for detoxification such as mobilization, immobilization or transformation of metals [68,69]. The immobilization mechanisms involve biosorption, biotransformation and bioaccumulation of metal ions by living microorganisms [70]. These bioremediation properties of yeasts can be exploited for the green synthesis of NPs to be applied in fields.

*Saccharomyces cerevisiae* was used for biosynthesis of AgNPs by biotransformation. Both the dried and fresh culture *S*. *cerevisiae* was used as the biocatalyst. More AgNPs were obtained from freshly cultured yeast than dried culture. The AgNPs were spherical with a size of 2–20 nm in diameter, and 5.4 nm sized particles were mostly found. AgNPs were found inside the cells, within the membrane of cells, attached to the cell membrane, and outside of the yeast cells [11]. A marine yeast *Yarrowia lipolytica* strain was used for the biosynthesis of AgNPs in a cell associated manner. This study suggested that the brown pigment (melanin) might be the possible reason for biomineralization of metallic ions [71]. *Pichia jadinii* was used for intracellular synthesis of AuNPs ranging from 1–100 nm. In this study, the growth and cellular activities of *P. jadinii* were controlled easily to regulate AuNPs size and shape [72]. The green synthesis AgNPs was obtained in an extracellular process by using *Candida utilis* NCIM 3469 with a size 20–80 nm [73]. In another study, *Saccharomy cescerevisiae* was capable of synthesizing copper NPs (CuNPs) extracellularly, where more than 70% of the particles were about 10–12 nm [74].

Several studies illustrate that viruses are considered to be a suitable group which serves as a biotemplate for material synthesis at the nanoscale to microscale [75]. Recently, material science researchers were using the viral NPs (VNPs) as templates or scaffolds for the synthesis of novel hybrid nanomaterials [76]. A number of plant viruses were employed as nano-factories because of their special structural integrity, easy manipulation and lower infectivity to human [76,77]. Furthermore, due to structural diversity, viral capsids are exploited as a biotemplate for material synthesis [78,79,80,81]. The viral NPs can be engineered genetically, chemically and also utilized as nano-templates at three levels of their structure [82]. The capsids of viruses are arranged by repeating protein subunits to form highly precise three-dimensional symmetrical structures with uniform shape and size [83,84].

The synthesis of nanomaterials using viruses is a clean, nontoxic and environmentally-friendly method which provides a broad range of sizes, shapes, compositions, and physicochemical properties [83,85]. In a study, a notorious plant pathogenic virus, *Squash leaf curl China virus* (SLCCNV) was used as biotemplate to fabricate silver and gold nanomaterials. The SLCCNV was exposed to HAuCl_4_ and AgNO_3_ precursors in presence of sunlight and quick (∼5 min) formation of SLCCNV-metallic-hybrid nanomaterials in an eco-friendly way was observed [86]. A wild type bacteriophage P22 was utilized for synthesis of cadmium sulfide (CdS) nanocrystal quantum dots on its ∼60 nm procapsid. The bacteriophage P22 shell possessed capsomers composed of hexameric and pentameric clusters. The pre-synthesized CdS quantum dots resemble the hexameric and pentameric patterns of assembly on the P22 shells, which might be due to interaction with particular protein pockets [87]. In another research, tobacco mosaic virus and bovine papilloma virus were used as additive materials with plant extracts *Avena sativa*, *Hordeumvulgare*, *Musa pradisiaca* and *Nicotiana benthamiana*. These two viruses promoted the reduction and increase the NPs number remarkably as compared to a control without virus [14,88]. These viral synthesized nanomaterials have a wide range of applications in biomedicine and serve as catalysts to biosensors [89]. Similarly, M13 bacteriophage can be used as versatile template for engineering various nanomaterials [90].Although a number of viruses and bacteriophages have been exploited for green synthesis of metallic NPs, no study is available regarding their application in the control of phytopathogens.

### 2.2. Nanoparticles from Microalgae

The synthesis of microalgae-based NPs, termed “phyconanotechnology”, has become an emerging area with wide scope in recent years. A large number of photoautotrophic microorganisms belong to microalgae which contain secondary metabolites, pigments and proteins [91,92]. These microorganisms can act as nano biofactories for synthesis of metallic NPs [93,94,95,96,97]. A number of methodologies have been developed for metallic NPs synthesis using microalgae from their corresponding aqueous salt solutions which can determine the size and shape of NPs with good quality. Synthesis of microalgae driven NPs can be obtained by using extracted biomolecules from disrupted cells of microalgae [92,98].

Microalgae can be exploited as an efficient bionanofactory, capable of producing metallic NPs by reducing various metal ions such as silver, gold, cadmium, and [98,99]. Both the live and died dried biomass of microalgae can be used to synthesize metallic NPs. Several microalgae such as *Chlorella vulgaris, Spirulina platensis*, and *Lyngbya majuscule* have been utilized for biosynthesis of AGNPs [93,100]. The biosynthesis of AgNPs extracellularly using a marine cyanobacterium, *Oscillatoriawillei* NTDM01 which reduced silver ions and stabilized the AgNPs by a secreted protein. The extracted biomolecules of *Chlorella vulgaris*, a single-celled green microalga was used to synthesize AgNPs [101]. In another study, living cells *Chlorella vulgaris* were incubated along with gold chloride solution and after incubation cells were harvested followed by centrifugation. NPs were detected inside intact cells by TEM and assigned to metallic gold by synchrotron-based X-ray powder diffraction and X-ray absorption spectroscopy. The sizes of the intracellular AuNPs were 40–60 nm in diameter [102]. Arsiya et al. reported the green synthesis of palladium NPs by *Chlorella vulgari*s aqueous extract [103]. The biomass was dried, powdered homogeneously, boiled in water and the crude extract was filtered. Aqueous solution of PdCl_2_ was mixed with crude filtrate of *Chlorella vulgari*s and solution color was changed to yellow dark brown indicating formation of PdNPs [103]. Marine microalgae such as *Chaetoceros calcitrans*, *Chlorella salina*, *Isochrysis galbana* and *Tetraselmis gracilis* were used to synthesize AgNPs [104]. A large number of studies of algal NPs are available but their use in phytopathogenic control is yet to be determined. A list of NPs have been synthesized from various microbes are given in Table 1.

### 2.3. Nanoparticles from Plant Extracts

Plants are well known for their ability to reduce metal ions on the surface and different organs or tissues at distance from the penetration sites of ions. The study of the accumulation of metal ions in plants suggested the transformation of metals to NPs [123]. For example, *Medicago sativa* and *Brassica juncea* grown withAgNO_3_ accumulated 12.4 wt. % and 13.6% wt. % silver, respectively, as AgNPs with a size of 50 nm [124]. In other studies, gold icosahedra of 4 nm in size were observed in *M. sativa* [125], and 2 nm of semi-spherical copper particles were observed in *Iris pseudacorus* [126] grown on the respective metal salt-containing substrates.

In recent years, various in vitro approaches have been developed in which plants extracts are being used as reducing agents for synthesis of NPs [123]. For this green synthesis of NPs, extracts of different plant species along with a variety of acids and metal salts, such as copper, gold, silver, platinum, iron have been used [127,128,129].The plant materials that have been used for NPs synthesis are more beneficial than microbial or chemical methods as there are no effects of microbes or hazardous chemical contamination. Moreover, it requires less energy and has easy and broader implications [130]. The green synthesis of NPs mediated by plant extracts involves the alleviation of metal ions [131] due to the presence of biomolecules such as phenols, terpenoids, ketones, carboxylic acids, aldehydes, enzymes, amides, and flavonoids [130,132,133,134,135]. The plant extracts prepared from their different parts such as roots, stems, barks, leaves, flowers, fruits and seeds have been used for green synthesis of NPs [136,137,138,139,140,141,142]. The plant extracts can function as reducing and stabilizing agents in the green synthesis of NPs [133].

A variety of green synthesized NPs from different plant species have been reported in last few years. For example, AgNPs have been synthesized from fruit extract of *Phyllanthus emblica* [130], leaves extract of *Citrus limon* [143], green tea (*Camellia sinensis*) [144], *Coffea Arabica* [145], neem (*Azadirachta indica*) [146], *Acalypha indica* [147], Aloe vera plant extract [148], latex of *Jatropha gossypifolia* [149], root extract of *Morinda citrifolia* [150], *Phoenix dactylifera* [151], inflorescence extract of *Mangifera indica* [152]. Zinc oxide NPs (ZnONPs) and titanium dioxide NPs (TiO_2_NPs) synthesized from extract of fresh lemon fruits (*Citrus limon*) showed antibacterial activity [133]. Zinc oxide NPs (ZnONPs) synthesized from extract of chamomile flower (*Matricaria chamomilla*), olive leave (*Olea europaea*) and red tomato fruit (*Lycopersicon esculentum*) showed antibacterial actions. Aqueous Rosemary extract was used to synthesize the MgO nano-flowers (MgONFs) having antibacterial potentials [132]. The quality, size and shape of these green synthesized NPs are dependent on various factors like plant extract concentrations and their compositions, metal salt concentration, reaction pH, reaction temperature [12,13,153]. Some NPs recently synthesized in a green way from plant materials used for plant pathogen management are listed in the Table 2.

## 3. Green Synthesized Metallic Nanoparticles for Control of Phytopathogens

**Figure 2:**
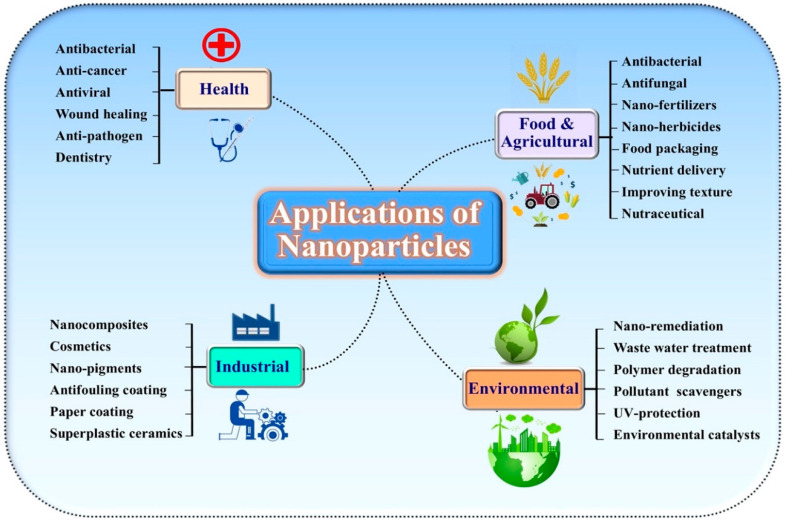
Application of nanoparticles in various fields.

NPs have been used in various fields including plant diseases control [130,151] (Figure 2). Generally, pesticides are used selectively to the pests of the crop plants without affecting on plants themselves along with the symbiotic flora, fauna and human beings [161]. However, the indiscriminate applications of these pesticides tend to be the serious public concerns and environmental issues [162]. Moreover, the occurrence and development of new pathogenic strains is a continuous problem, the chemical application is expensive and also not always effective. Recently, the progress in the area of green synthesis has inspired scientists and researchers to utilize its potentials against the pathogenic microorganisms. Biosynthesized metallic NPs such as silver, copper, gold, and zinc, have been reported to act against both Gram-positive and Gram-negative bacteria such as *B. subtilis*, *E. coli*, and *Staphylococcus aureus*. These NPs showed inhibitory actions against some pathogenic fungi including *A. niger*, *F. oxysporum*, *A. fumigatus*, and inhibitory effects against other pathogenic microbes [163]. The silver NPs have received more attention due to “green synthesis” from plants, bacteria, fungi or yeasts [164]. Fouda et al. [43] observed the antimicrobial activity of green synthesized AgNPs from endophytic *Streptomyces* against four plant pathogenic fungi represented by *Alternaria alternata*, *Fusarium oxysporum*, *Pythiumultimum*, and *Aspergillus niger*. The green synthesized ZnONPs and TiO_2_NPs with lemon fruit extract at room temperature showed their antibacterial activities against *Dickeya dadantii*, a causal bacterium of sweet potato stem and root rot disease [133]. The MgO and MnO_2_ NPs synthesized using chamomile flower extract showed activity against *Acidovorax oryzae* strain RS-2, a bacterium causing bacterial brown stripe disease of rice. Nanotechnology has potential prospects in plant pathogenic management in various ways. The most commonly used method is the direct application of NPs to seeds or foliar spray against the plant pathogens, which are more greatly suppressed compared to chemical pesticides. In another method, nanomaterials are used as carrier materials of chemical active ingredients for controlled release of pesticides [1]. Here, we list the green synthesized metallic NPs with their size, shape, and targeting plant pathogens in Table 1 and Table 2.

## 4. Mechanisms of Action of Nanoparticles against Phytopathogens

Although green synthesized metallic NPs have been studied for their potentials against phytopathogens, the exact mode of actions of NPs is not completely understood [165]. To date, some mechanisms have been reported, such as protein dysfunction (e.g., oxidation of cysteine in Fe-binding site, destruction of Fe–S cluster, exchange of catalytic metal, and exchange of structural metal), production of reactive oxygen species(ROS) and antioxidant depletion, impaired membrane function (e.g., membrane damage, loss of membrane potential), interference with nutrient uptake (e.g., inhibition of Fe (III) transporter gene expression) and genotoxicity (e.g., double-strand breaks) [166]. These mechanisms may not operate individually, but function in combination against various phytopathogens [52,166].

The adhesion of NPs with microbial cell membrane occurs due to the electrostatic attraction between the negatively charged cell membrane of microbes and NPs with positive or low negative charges. The morphological structures of the membrane are disturbed by the NPs and the membrane depolarization causes the disruption of membrane permeability and respiratory actions, and ultimately damages the cell structures, leading to cell death. This disruption of cell structure leads to the leakage of internal cell content including proteins, enzymes, DNA, and metabolites. In addition, the NPs may cause irregular pits on the microbial cell wall that facilitate NPs’ entry into periplasmic space and inside the cells. NP actions on membrane damage and pit formation on the cell surface can be observed by using TEM and SEM [14]. Masum et al. observed the green synthesized AgNPs effects on *Acidovorax oryzae* strain RS-2 using TEM and showed highly ruptured cell walls, leakage of cytoplasmic and nucleic contents, swollen structure leading to bacterial death [112]. In another study, biosynthesized AgNPs were applied to *Fusarium graminearum* strain PH-1 and antifungal activities of distortion of hyphae and damaging cell walls were observed by both SEM and TEM [20]. Similar effects of AgNPs on several other phytopathogenic fungi such as *Alternaria alternata*, *Botrytis cinera* and *Trichosporon asahii* were also observed [167].

The toxicity of NPs may be caused by the formation of ROS [143]. Free radicals can damage the cell wall and various biomolecules such as proteins, lipids and DNA. DNA damages, such as mutations, deletions, single-strand breaks, double-strand breaks, and cross-linking with proteins, may occur [168]. Zhang et al. revealed that damaging cell membrane and generating ROS are involved in the antibacterial mechanisms [169]. They observed damage of cell membrane and AgNPs inside *Azotobacter vinelandii* cells using TEM and revealed AgNP-induced hydroxyl radicals inside bacterial cells using electron spin resonance.

## 5. Environmental Consequences of Metallic Nanoparticles

**Figure 3:**
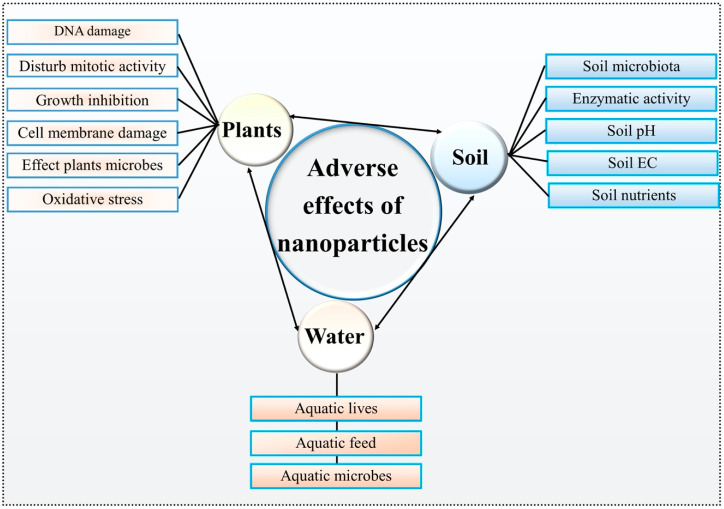
Adverse effects of metallic NPs on major elements (plants, soil and water) in agroecosystems.

Several studies have revealed that NPs can be used to manage or control plant pathogens [5,6]. Besides this benefit, there may be some toxic or adverse effects of these NPs on various components of the environments (Figure 3). The NPs that have been used for plant pathogen management may be dispersed from the crop lands to the soil, water and atmosphere. The dispersion may take place through leaching, surface run-off by rain, and transport by air current or trophic transfer [170]. Different studies on this subject have suggested that NPs may be absorbed by microbes in the soils, sediments and plant roots. Later, these NPs are migrated from roots to other parts of the plants, and accumulation occurs [171]. Shifting of NPs from one trophic level to another trophic level takes place as the microbes, plant products or their waste materials are utilized or consumed by various organisms such as protozoa, arthropods, annelids, mollusks, fish, insects, birds and mammals [172,173]. This demonstrates that the adverse effects may be inherited by their offspring [174]. This scenario has also been observed in marine organisms [175] and also in food chains of plant-herbivore-carnivore [172,173,176,177]. Therefore, a standard application of nanomaterials in crop plants is needed for safe and sustainable use of nanotechnology in agriculture. For the management of plant pathogens, the application of NPs should be accompanied with the knowledge of possible risks for direct or indirect application to crop plants, and the ecosystem where the crop plants have interacted with microorganisms, animals or human beings. Below, we discuss the toxicity or adverse effects of metallic NPs on plants and beneficial microbes in more detail.

## 6. Toxicity of Metallic Nanoparticles on Plants

The ultra-small size of the NPs is the main cause to facilitate the inhibitory actions against the various plant pathogens. Indeed, this is the similar basic phenomenon of adverse effects of NPs on the surrounding environment, animals, plants, human and beneficial microorganisms [1]. The NPs can be applied to both the above-ground parts and below-ground parts of the plants to manage the plant diseases. In addition, seeds may be dipped into biosynthesized NPs solution to manage the seedling diseases and the pathogenic attack at a later stage. The applied NPs may inhibit the plant pathogens locally in various plant parts and also may become translocated throughout the plant system, thus reducing the occurrences of the disease. However, diversified NPs were subjected to plant toxicity due to their uptake, translocation and accumulation in the plant cells or organs [178]. The NPs uptake and translocation depends on multiple factors such as properties of NPs (size, composition and surface area), dose of application, delivery techniques, and plant species. The bioaccumulation may alter the plant physiology affecting the growth and development of plants [179]. Metal NPs are known to be highly toxic, even at low concentrations, although the NPs are rarely dispersed in the environment [180]. Research into NPs toxicity in crop plants is still in its infancy, but the application of innovative agro nanotechnology tools and products is crucial for agricultural development [181]. In most studies, for cultivated crop plants, such as tomato, wheat, onion, and zucchini, the excess metallic NPs may trigger oxidative burst through the interference of electron transport chain. It also can impair the ROS detoxifying process, resulting in genotoxic implications [182,183,184,185]. As a result, production of secondary metabolites and phytohormones are affected, and retarded plant growth occurs [186].

The toxicity and adverse effects that have been reported in plants include constraints in water transport in plant systems, decrease in the growth hormone production, metabolic disorder, oxidative stress, chromosomal abnormalities decreased growth, deviation in transcriptional profile of several genes, raising susceptibility to natural toxins (e.g., Arsenic) [170].

## 7. Toxicity of Metallic Nanoparticles on Beneficial Microbes Associated with Plants

Microbes are epiphytically and endophytically associated with plants, in rhizosphere soil, and bulk soil near the plant root system, and may promote plant growth through the production of phytohormone (e.g., auxin) and siderophore, nitrogen fixation, and phosphate solubilization [187]. NPs applied to plants and soils may be toxic to these beneficial microbes in the same manner as they are to the plant pathogens. The effects on soil microbial community can be assessed by the observation of respiration and enzymatic activities in soils [188]. For example, CuONPs and TiO_2_NPs reduced biomass, total phospholipid fatty acids and activities of enzymes, such as urease, phosphatase and dehydrogenase, of soil microbiota in a flooded rice field. The principle component analysis of phospholipid fatty acids and diversity indices indicated that CuONPs affected the microbial composition and diversity [189]. Similarly, CeO_2_, Fe_3_O_4_, TiO_2_ and ZnONPs had effects on bacterial community and activities of enzymes (catalase, invertase, phosphatase, and urease) in saline alkali and black soils [190]. ZnONPs and CeO_2_NPs reduced thermogenic metabolism, numbers of *Azotobacter*, phosphate solubilizing and potassium solubilizing bacteria, and enzyme activities in soil; TiO_2_NPs reduced the enzymatic activities and abundance of soil bacteria [191].

NPs can affect plant-associated and soil microbes beneficial to plants at the time for phytopathogen control. Chavan and Nadanathangam evaluated the effects of AgNPs and ZnONPs on nitrogen fixers (*Rhizobium leguminosarum* MTCC 10096, *Sinorhizobium meliloti* clsxc_S_SNF, *Azotobacter chroococcum* clsxc_A_FLNF), phosphate solubilizers (*Arthrobacter* sp. MTCC 8160, *Bacillus* sp. clsxc_ NPS, *Serratia marcescens* MTCC 7642, *Pantoea dispersa*clsxc_PSD) and biofilm formers (*Bacillus subtilis* MTCC 441, *Bacillus* sp. clsxc_TYA, *Pseudomonas aeruginosa* MTCC 7763, *Klebsiellapneumonia* clsxc_AZ2). AgNPs showed bactericidal effects at lower concentrations of 2–22 µg/mL whereas ZnONPs showed a bacteriostatic effects up to concentrations of 3000 µg/mL in some cases. Moreover, AgNPs significantly changed the bacterial community in soil [192]. Another study assessed the toxicity of ZnONPs and CeO_2_NPs against *Sinorhizobium meliloti*, a symbiotic alfalfa inhabiting bacterium using advanced microscopic and spectroscopic techniques. ZnONPs were more toxic than CeO_2_NPs according to the viable cell counts and inhibition of growth dynamics [193]. Microbial community analysis using culture-dependent and independent methods showed that ZnONPs altered the microbial community in soil [194].

## 8. Conclusions and Perspectives

Recent studies show that the biogenic or green-synthesized metallic NPs using microbes and plants without hazardous chemicals are promising in control plant pathogens including bacteria, fungi, oomycetes, and viruses. On the other hand, the biogenic metallic NPs have toxic potentials to plants and plant-associated beneficial microbes and ultimately to human beings. Therefore, the positive and negative effects of these NPs on agriculture and environments should be ascertained before their commercial use in plant disease management in the field.

To achieve the green control of plant diseases with the green-synthesized NPs, we should pay attention to the following aspects in the near future. (1) AgNPs is the major metallic NPs tested on plant pathogens and shows toxicities to plants and plant-associated microbes. Less phytotoxic metals, such as Zn, Fe, Mg, and Mn, should be tested. (2) Minimum inhibitory concentration of metallic NPs on pathogens should be determined in vitro and in vivo. (3) Metallic NP effects on plant growth and development at the working concentration should be determined. (4) Metallic NPs at the working concentration in vivo should be less toxic to other plant-associated microbes than the target pathogens; Metallic NP effects on plant microbiota should be determined. (5) The dynamic of the metallic NPs and their effects on soil microbiota should be determined. Few studies on plant disease control in fields by metallic NPs have been done. Future studies should determine the efficiency of metallic NPs in fields along with the assessment of their potential risks to environments and human health.

## Figures and Tables

**Table 1:** Microbes mediated synthesized metallic NPs against plant pathogens.

Microbes	Sources of Isolation	MetalNPs	Features		Major Application	Plant Disease Management		
			Size (nm)	Shape		Pathogen	Host	References
**Bacteria**								
*Pseudomonas rhodesiae*	Rhizospheric soil of cotton	Ag	20–100	Spherical	Antibacterial agent	*Dickeyadadantii*	Sweet potato	[8]
*Bacillus siamensis*	*Coriandrumsativum*	Ag	25–50	Spherical	Antibacterial agent	*Xanthomonas oryzae* pv. *oryzae*	Rice	[105]
*Bacillus* *cereus*	Wastewater contaminated soil	Ag	18–39	Spherical	Antibacterial agent	*Xanthomonas oryzae* pv. *oryzae*	Rice	[18]
*Pseudomonas poae*	Garlic	Ag	20–45	Spherical	Antifungal agent	*Fusarium graminearum*	Wheat	[20]
*Bacillus* sp.	Soil	Ag	7–21	Spherical	Antifungal agent	*Fusarium oxysporum*	Tomato	[106]
*Serratia* sp.	Soil	Ag	10–20	Spherical	Antifungal agent	*Bipolaris sorokiniana*	Wheat	[107]
*Stenotrophomonas* sp.	Soil	Ag	12	Spherical	Antifungal agent	*Sclerotium rolfsii*	Chickpea	[28]
*Pseudomonas* sp., and *Achromobacter* sp.	Rhizospheric soil of chickpea	Ag	20–50	Spherical	Antifungal agent	*Fusarium oxysporum* f. sp. *ciceri*	Chickpea	[108]
*Aeromonas hydrophila*	Missing	ZnO	57–72	Crystalline		*Aspergillus flavus*	Maize	[36]
*Streptomyces* spp.	*Oxalis corniculata* leaves	CuO	78–80	Spherical	Antifungal agent	*Alternaria alternata*, *Fusarium oxysporum*, *Pythium ultimum*, and *Aspergillus niger*	Multiple crops	[42]
*Streptomyces capillispiralis*	*Convolvulus arvensis* leaves	Cu	4–59	Spherical	Antifungal agent	*Alternaria* spp., *Aspergillus niger*, *Pythium* spp., and *Fusarium* spp.	Multiple crops	[109]
*Streptomyces griseus*	Rhizospheric soil of tea	Cu	5–50	Spherical	Antifungal agent	*Poria hypolateritia*	Tea	[110]
*Bacillus thuringensis*	Soil	Ag	10–20	Polymorphic	Antiviral agent	Sun hemp rosette virus	Cluster bean	[111]
*Bacillus licheniformis*	Soil	Ag	77–92	Polymorphic	Antiviral agent	Bean yellow mosaic virus	Faba bean	[112]
**Fungi**								
*Trichiderma hazarium*	Tomato	Ag	11–13	Spherical	Antifungal agent	*Helminthosporium* sp., *Alternaria alternata, Phytophthora arenaria,* and *Botrytis* sp.	Multiple crops	[113]
*Aspergillus niger*	Grape	Ag	10–100	Spherical	Antifungal agent	*Penicillin digitatum*, *Aspergillus flavus*, and *Fusarium oxysporum*	Multiple crops	[114]
*Penicillium duclauxii*	Corn seeds	Ag	3–32	Spherical	Antifungal agent	*Bipolaris sorghicola*	Sorghum	[115]
*Setosphaeria rostrata*	*Solanum nigrum* leaves	Ag	2–50	Spherical	Antifungal agent	*Aspergillus niger*, *Rhizoctonia solani*, *Fusarium graminearum,* and *Fusarium udum*	Multiple crops	[116]
*Trichoderma longibrachiatum*	Cucumber	Ag	1–25	Spherical	Antifungal agent	*Alternaria alternata, Pyricularia grisea, Fusarium verticillioides,* *Helminthosporium oryzae, and Penicillium glabrum*	Multiple crops	[117]
*Trichoderma harzianum*	Soil	Ag	20–30	Spherical	Antifungal agent	*Sclerotinia sclerotiorum*	Cabbage	[118]
*Guignardia mangiferae*	Leaves of medicinal plants	Ag	5–30	Spherical	Antifungal agent	*Rhizoctonia solani*	Rice	[119]
*Arthroderma fulvum*	Soil	Ag	13–18	Spherical	Antifungal agent	*Aspergillus flavus*	Maize	[120]
*Aspergillus versicolor*	Soil	Ag	5–39	Spherical	Antifungal agent	*Sclerotinia sclerotiorum* and *Botrytis cinerea*	Strawberry	[121]
*Fusarium solani*	Wheat grain	Ag	5–30	Spherical	Antifungal agent	*Fusarium* spp., *Aspergillus* spp., *Alternaria* spp. and *Rhizopus stolonifer*	wheat, barley and corn	[122]
*Cephalosporium* sp. and *Trichoderma* sp.	Rhizospheric soil of chickpea	Ag	20–50		Antifungal agent	*Fusarium oxysporum* f. sp. *ciceri*	Chickpea	[108]

**Table 2:** Plants mediated synthesized metallic NPs against plant pathogens.

Plants	Plant Parts Used	Metal NPs	Features		Plant Disease Management		References
			Size (nm)	Shape	Pathogen	Host	
*Citrus limon*	Fruits	ZnO and TiO2	20–200	Polymorphic	*Dickeya dadantii*	Sweet potato	[133]
*Phyllanthu semblica*	Fruits	Ag	20–93	Spherical	*Acidovorax oryzae*	Rice	[130]
*Rosmarinus officinalis*	Flowers	MgO	<20	Flower	*Xanthomonas oryzae* pv. *oryzae*	Rice	[132]
*Matricaria chamomilla*	Flowers	MgO and MnO2	9–112	Disk-shapedSpherical	*Acidovorax oryzae*	Rice	[134]
*Matricaria chamomilla*	Flowers	ZnO	50–192	Crystalline	*Xanthomonas oryzae* pv. *oryzae*	Rice	[7]
*Olea europaea*	Leaves	ZnO	41–124	Crystalline	*Xanthomonas oryzae* pv. *oryzae*	Rice	[7]
*Lycopersicon esculentum*	Fruits	ZnO	66–133	Crystalline	*Xanthomonas oryzae* pv. *oryzae*	Rice	[7]
*Piper nigrum*	Stem	Ag	9–30	crystalline	*Erwinia cacticida*	Watermelon	[154]
*Citrus maxima*	Fruits	Ag	11–13	Spherical	*Acidovorax oryzae*	Rice	[155]
*Artemisia absinthium*	Leaves	Ag	5–100	Spherical	*Phytophthora parasitica*	Citrus	[156]
*Trachyspermum ammi*	Leaves	Ni	68	Missing	*Colletotrichum musae*	Banana	[157]
*Abelmoschus esculentus*	Seed	Au	45–75	Spherical	*Puccinia graminis* pv. *tritci*	Wheat	[158]
*Parthenium hysterophorus*	Leaves	ZnO	28–84	Spherical andHexagonal	*Fusarium culmorum*	Barley	[159]
*Syzygium aromaticum*	Bud	Cu	15	Spherical	*Aspergillus niger*, *Aspergillus flavus*, and *Penicillium* spp.	Multiple crops	[160]
